# Hoffa’s fat pad resection during total knee arthroplasty does not affect functioning and gait: a double-blind randomized clinical trial

**DOI:** 10.1007/s00402-024-05503-2

**Published:** 2024-08-28

**Authors:** Joyce L. Benner, Kirsten D.S. Boerma-Argelo, Myrthe D. Simon-Konijnenburg, Marco J.M. Hoozemans, Bart J. Burger

**Affiliations:** 1 Department of Orthopaedic Surgery, Centre for Orthopaedic Research Alkmaar (CORAL), Northwest Clinics, Wilhelminalaan 12, Alkmaar, 1815 JD The Netherlands; 2 Department of Human Movement Sciences, Faculty of Behavioral and Movement Sciences, Amsterdam Movement Sciences, Vrije Universiteit Amsterdam, Van der Boechorststraat 9, Amsterdam, 1081 BT The Netherlands

**Keywords:** Total knee arthroplasty, Fat pad, Functional outcome, Gait analysis

## Abstract

**Introduction:**

Hoffa’s fat pad is considered a source of anterior knee pain and may limit prosthetic knee function. Resection of Hoffa’s fat pad in total knee arthroplasty (TKA), however, is controversial, and little is known about the functional outcomes including gait quality. This double-blind randomized controlled trial (i) compared functional recovery between TKAs where Hoffa was resected or preserved, and (ii) compared recovery of self-reported function with objective (gait-related) outcomes.

**Materials and methods:**

Eighty-five patients (age 66.4 ± 8.0 years, 47% women) scheduled to undergo TKA for primary osteoarthritis were randomly assigned to either fat pad resection or preservation. Subjective measures of functioning were assessed at baseline, 6 weeks, 3 months, and 12 months postoperatively and included the Knee Injury and Osteoarthritis Outcome Score (KOOS), Kujala, and visual analog scale (VAS) for pain. Objective measures of functioning were assessed at baseline, 3 months, and 12 months postoperatively and included instrumented range-of-motion and gait analysis. Longitudinal analyses (generalized estimating equations) were used to compare recovery between groups, and chi-square tests compared attainment of minimal clinical important difference (MCID) and patient acceptable symptom state (PASS). Finally, correlation analyses explored associations between subjective and objective recovery in function.

**Results:**

Resection patients showed poorer improvement in KOOS quality of life in the first 6 weeks (B=–10.02, 95% confidence interval (CI) [-18.91, -1.12], *p* = .027), but stronger improvement in knee extension after 3 months (B = 3.02, 95%CI [0.45, 5.60], *p* = .021) compared to preservation patients. Regarding MCID or PASS, no differences were noted between groups at 3 and 12 months (all *p* > .05). Subjective function substantially improved in the first 3 months, while objective outcomes improved only between 3 and 12 months. Moderate to strong correlations were identified between changes in knee flexion and gait with Kujala and KOOS in the resection but not in the preservation group.

**Conclusions:**

Similar functional outcomes were achieved after TKA with or without resection of Hoffa’s fat pad. Hence, removing the fat pad to promote surgical exposure will not affect functional outcomes including gait quality. Functional recovery of objective outcomes was not always consistent with subjective recovery, suggesting that both self-reported as well as objective, gait-related outcomes may provide meaningful information on functional recovery following TKA.

**Trial registration:**

This clinical trial was prospectively registered under the Netherlands Trial Registry (# NL3638). This registry has recently been replaced by the Dutch Trial Registry where this study can be accessed via https://onderzoekmetmensen.nl/en/trial/20994.

## Introduction

Total knee arthroplasty (TKA) is one of the most effective interventions for the treatment of end-stage knee osteoarthritis, with satisfaction rates up to 90% one year post-surgery [1]. Anterior knee pain and limitations in daily functioning are reported as common reasons for dissatisfaction [2]. Hoffa’s fat pad is suggested to contribute to developing anterior knee pain [3], and its resection during TKA could reduce pain and aid in functional recovery at no cost and minimal risk.

Hoffa’s fat pad is often resected during TKA as it enhances surgical exposure, but literature on its clinical advantages offers contradictory findings [4]. Hoffa’s fat pad is thought to play a role in the inflammatory process and contains nociceptive fibers, making it an obvious source of knee pain [3]. However, increased anterior knee pain has been reported for both resection as well as preservation [5, 6]. Furthermore, some studies indicate that resection of Hoffa’s fat pad leads to increased postoperative stiffness, while others suggest no functional differences compared to preservation of the fat pad [7]. Recently, Sun et al. [4]. concluded in a meta-analysis of randomized controlled trials that resection was not superior to preservation concerning knee pain, passive range-of-motion, or other clinical outcomes. Walker et al. [8], however, found some differences in pain outcomes and knee flexion in their meta-analysis. Judging from these clinical outcomes, the debate on resection of Hoffa’s fat pad remains.

Fewer studies have, however, focused on functional outcomes following fat pad resection [6, 9], which are known to strongly correlate with patient satisfaction [10]. Usually, patient-reported outcome measures (PROMs) are used to assess functional outcomes, however, their clinical value is limited due to their subjectivity, ceiling effects, and pain dominance. Alternatively, performance-based tests have been suggested to objectively capture physical functioning, including gait analysis [11]. Gait analysis has been recommended as an additional means to evaluate functional outcome after TKA [11]. However, concerns remain about the discrepancy between PROMs and gait-related measures for the assessment of physical functioning in orthopedic populations [13–15]. Recovery often appears faster based on PROMs than when measured by objective assessments [16]. Objective assessment may be of added value when determining whether a particular intervention, like resection of Hoffa’s fat pad in TKA, is more beneficial regarding functional outcomes.

Literature to date remains contradictory on the impact of Hoffa’s fat pad resection on pain and stiffness, and little is known about recovery and outcomes of functioning. Therefore, this study aimed to (i) compare subjective and objective functional recovery up to one year post-surgery between TKA patients with or without resection of Hoffa’s fat pad, and (ii) compare the objective outcomes following TKA with PROMs in both resection and preservation patients to determine whether (advanced) objective assessments provide additional information about functional outcome beyond standard clinical tools in the context of Hoffa’s fat pad resection.

## Materials and methods

### Study design

This was a single-center, double-blind, randomized controlled trial with a parallel design conducted in a large teaching hospital and reported according to the CONSORT guidelines. All study procedures were approved by the Medical Ethical Committee Noord-Holland (M012-009; NL39455.094.12) and the institutional review board (L012-038). All patients provided written informed consent.

### Study population

Patients with radiographically confirmed Kellgren-Lawrence classification grade 3–4 knee osteoarthritis on the waiting list for a primary TKA were invited. Inclusion criteria were age 30–80 years and American Society of Anesthesiologists (ASA) status I-III. Exclusion criteria were a contralateral hip/knee arthroplasty or ipsilateral hip arthroplasty, rheumatoid arthritis, insulin-dependent diabetes, lack of understanding of the Dutch language, or any neurological or muscle disorders limiting gait assessment.

An a priori sample size calculation determined that 41 patients per group were needed to detect an expected difference of 5 degrees (SD 8.0) in knee flexion range-of-motion between the group with and without resection of Hoffa’s fat pad [17], given a power of 0.8 and an alpha of 0.05.

### Randomization & blinding

Shortly before surgery, included patients were randomized in a 1:1 ratio by a trained research coordinator to undergo TKA with (intervention) or without resection (control) of Hoffa’s fat pad. Randomization was computer-generated, and allocation was stored in an opaque, sealed envelope and concealed from the researchers assessing patients. Envelopes were opened during surgery and allocation was shown to the surgeon only. Patients were not able to notice whether their fat pad was resected, either by visual or tactile inspection. Hence, both the researchers performing the assessments and the patients were blinded for treatment; only the surgeon was aware of the allocation. All patients received a Genesis II fixed-bearing, posterior-stabilized, or cruciate-retaining TKA including patellar component (Smith & Nephew, London, United Kingdom). Surgeries were performed by three orthopedic surgeons using the parapatellar approach and following standardized peri- and postoperative protocols. Adverse events due to the study intervention were not expected, only events related to the surgery or anesthesia. Any undesirable, adverse events occurring during the study, whether or not related to the surgery, were followed until abated and documented.

### Outcome measures

Self-reported pain and physical function were evaluated at baseline and at 6 weeks, 3 months, and 12 months postoperatively using the Knee injury and Osteoarthritis Outcome Score (KOOS) [18], the Kujala score [19], and the visual analog scale (VAS) for pain. The KOOS is subdivided into five subscales (activities of daily living (ADL), pain, quality of life (QoL), symptoms, and sports and recreation), with scores ranging from 0 (worst possible) to 100 (best possible). The KOOS sports and recreation subscale has negligible value for evaluating TKA outcomes, as most patients are not normally engaging in activities measured by this subscale (e.g., running or jumping) and was therefore not analyzed in this study.

Objective functioning was evaluated by active and passive knee range-of-motion using photo analysis [20] at baseline, 3 months, and 12 months postoperatively. Markers were attached to the patient’s skin at the rotation centers of the ankle, knee, and hip, visually estimated from a sagittal view. Patients bent and straightened their involved leg as far as possible without any assistance while in a supine position (active), or the research assistant tried to bend and stretch the knee as far as the patient allowed (passive). The marker positions in full flexion and full extension were recorded using a standard digital photo camera positioned at a height equal to the patient’s hip and at a distance sufficient to make all markers visible on-screen. Photographs were analyzed using a custom script in Matlab (R2021b; MathWorks, Natick, USA) to determine the maximal knee flexion and extension angles.

Gait quality was measured at baseline and 12 months using a tri-axial trunk-worn accelerometer (Dynaport Hybrid, range − 8 to 8 g, McRoberts, The Hague, The Netherlands; or MetaMotionR, range − 8 to 8 g, MbientLab, San Francisco, USA). Accelerations were measured in the anterior-posterior (AP), medial-lateral (ML), and vertical (VT) directions with the sampling rate set to 100 Hz. The accelerometer was placed at the patient’s lower back at the sacrum level using a velcro belt. Patients were instructed to walk 50 m back and forth at their self-selected pace in the outpatient clinic hallway. Raw accelerometer data were analyzed using a custom Matlab (R2021b; MathWorks, Natick, USA) script [21]. Gait speed, stride length, gait symmetry (harmonic ratio), and stride regularity in all directions were calculated [15], with higher values indicating better gait quality. Patients were also instructed to walk 10 m four times whilst being timed to calculate their preferred walking speed (10 m-PWS). The 10 m-PWS was determined at baseline, 3 months, and 12 months postoperatively.

### Statistical analysis

To determine the effects of TKA and the additional effects of Hoffa’s fat pad resection compared to preservation, generalized estimating equations (GEE) analyses with exchangeable correlation structures were performed according to the intention-to-treat principle [22]. First, linear models were made for each outcome measure with solely time as predictor variable to determine the repeated effects of TKA in general. Then, overall models were made with group allocation as a predictor to determine the overall intervention effects, while correcting for baseline differences. Finally, between-group differences for the follow-up intervals (∆T0-T6w, ∆T6w-T3m, ∆T3m-T12m) were assessed by adding time and an interaction term between group and time to the overall models. Regression coefficient B, *p*-value, and the 95% confidence interval (CI) for the crude models are reported. The B of the overall model represents the between-group difference for all measurements, whereas the B at the specified interval represents the between-group difference for that interval. The control group was the reference group in all models. Missing data were not imputed in any analysis.

Clinical improvements from baseline to 3 months (∆T0-T3m) and from baseline to 12 months (∆T0-T12m) were calculated, and frequencies of patients with change scores exceeding the minimal clinical important difference (MCID) were determined. Furthermore, frequencies of patients reaching the Patient Acceptable Symptom State (PASS) were determined at 3 and 12 months. Then, frequencies of patients reaching MCID and PASS were compared between groups using chi-squared tests. The MCID and PASS values, respectively, for each of the KOOS subscales used were 16 and 83 (ADL), 18 and 84 (pain), 17 and 66 (QoL), and 7 and 80 (symptoms) [23, 24]. For VAS pain, respectively, 18 and 25 were used [24], and for both 10 m-PWS and gait speed, respectively, 0.13 m/s and 1.20 m/s were used [25, 26]. For all other outcomes, no reliable MCID or PASS values have been established specifically for TKA patients in literature and thus were not evaluated.

To determine the association between PROMs and objective measures in both groups, Pearson’s correlations for the different change scores (i.e., ∆T0-T3m, ∆T3m-T12m, and ∆T0-T12m) were calculated. Values of 0.5 or higher were considered strong associations, 0.3–0.5 were considered moderate, and 0.1–0.3 were considered weak associations [27]. All analyses were performed using SPSS version 28.0 (IBM Corp, Armonk, USA) with *p-*values < 0.05 being considered significant.

## Results

**Fig. 1:**
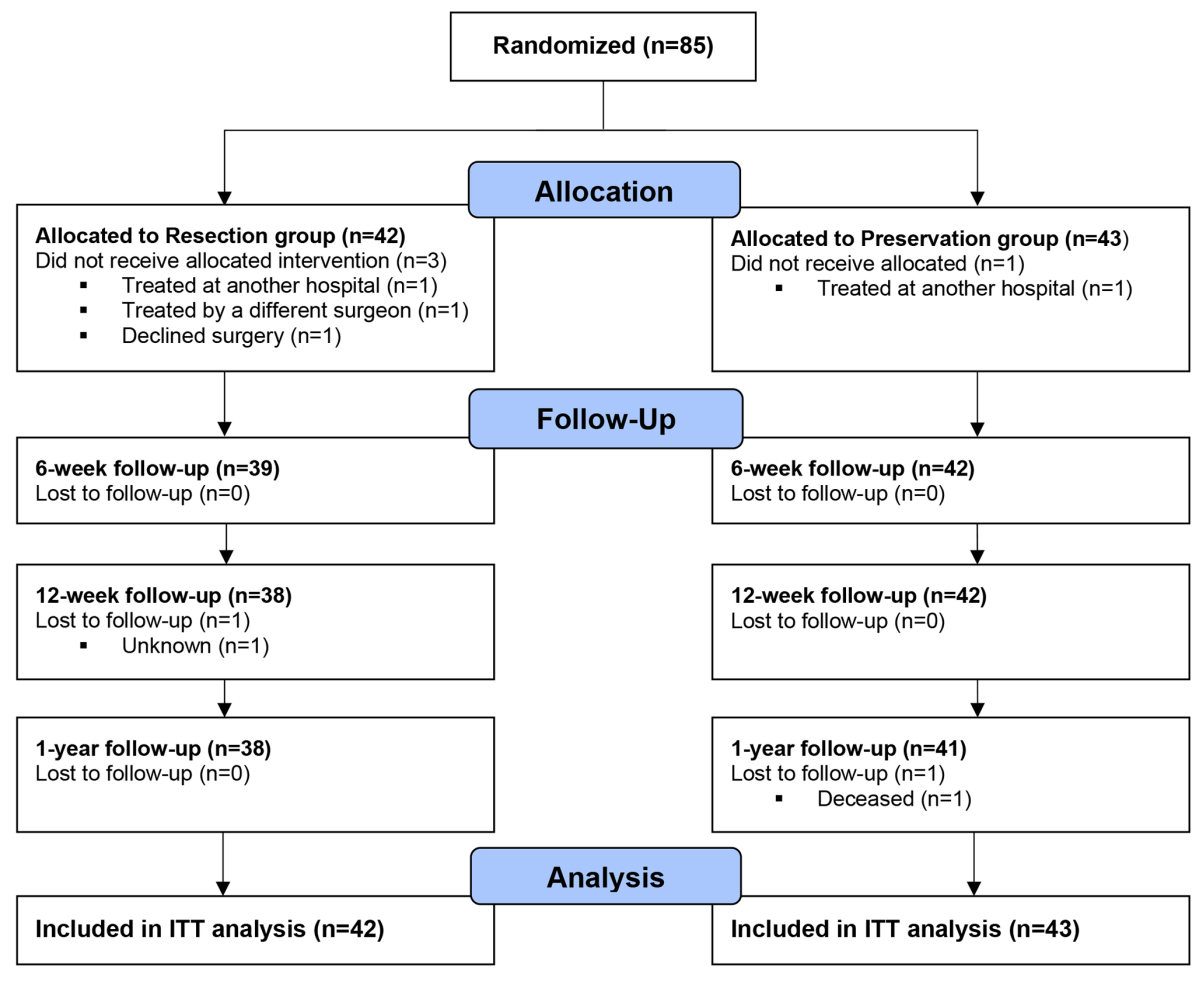
CONSORT flow diagram showing participant flow

In total, 85 patients were randomized to either the resection (*n* = 42) or preservation (*n* = 43) group, of whom 79 (93%) completed the study (Fig. 1). Four patients did not receive the TKA (in our institution), one patient died during follow-up, and one patient was lost to follow-up for reasons unknown. During the study, six adverse events were documented, all unrelated to the interventions or surgery (e.g., contralateral surgery, myocardial infarction, decease). No significant differences were observed between the intervention and control groups concerning baseline or intraoperative characteristics (Table 1). Furthermore, no statistically significant differences were found in baseline PROMs or objective outcomes.

**Table 1:** Baseline characteristics for intervention and control groups

	Intervention group (resection, *n* = 42)	Control group (preservation, *n* = 43)	*p*-value
Men, *n* (%)	17 (41%)	23 (54%)	0.229
Age, years	67.2 ± 8.4	65.7 ± 7.6	0.370
BMI, kg/m^2^	27.7 ± 5.1	28.7 ± 3.9	0.300
Prosthesis design			0.623
Cruciate-retaining	16 (41%)	15 (36%)	
Posterior-stabilized	23 (59%)	27 (64%)	
ASA classification, *n* (%)			0.331
I	12 (31%)	8 (19%)	
II	23 (59%)	26 (62%)	
III	4 (10%)	8 (19%)	
Smoking, *n* (%)	5 (12%)	6 (14%)	0.778
Surgery time, minutes	75.2 ± 16.6	73.3 ± 18.6	0.625
Length of stay, days	3.3 ± 1.9	2.9 ± 1.4	0.215

*n*, number of patients; BMI, body mass index; kg, kilograms; m, meters; ASA, American Society of Anesthesiologists

### Intervention effects on subjective outcomes

PROMs scores over time are shown in Table 2, and longitudinal analysis results are presented in Table 3. Regardless of group allocation, significant improvements were found for all the PROMs from baseline to each subsequent postoperative measurement, except for the Kujala between baseline and 6 weeks (*p* = .156). No overall intervention effects were found for any of the PROMs. On specific intervals, only a significant intervention effect was found for the KOOS QoL subscale in the first 6 weeks in favor of the control group (B − 10.02, 95%CI[ − 18.91, − 1.12], *p* = .027), meaning that the control group showed greater improvement in the first 6 weeks compared to the intervention group. No differences in proportions of patients reaching MCID were noted, nor were differences in PASS found between groups (Table 2).

**Table 2:** Subjective outcomes for intervention and control groups

		Intervention group (resection, *n* = 42)	Control group (preservation, *n* = 43)
Kujala	T0	46.1 ± 5.6	45.6 ± 5.9
	T6w	45.6 ± 14.7	48.5 ± 10.6
	T3m	56.5 ± 15.0	56.5 ± 15.6
	T12m	74.1 ± 19.7	74.7 ± 13.7
KOOS, ADL	T0	47.3 ± 8.9	47.5 ± 8.9
	T6w	63.8 ± 13.5	67.6 ± 17.9
	T3m	71.7 ± 17.4	74.9 ± 15.2
	T12m	83.5 ± 16.1	83.6 ± 16.8
	MCID ∆T0-T3m	23 (72%)	30 (79%)
	MCID ∆T0-T12m	27 (87%)	33 (85%)
	PASS T3m	12 (34%)	13 (34%)
	PASS T12m	23 (68%)	28 (72%)
KOOS, pain	T0	44.3 ± 9.1	42.3 ± 9.6
	T6w	57.6 ± 15.4	62.2 ± 20.6
	T3m	68.6 ± 19.5	69.7 ± 17.7
	T12m	85.2 ± 16.4	84.2 ± 17.8
	MCID ∆T0-T3m	20 (61%)	24 (63%)
	MCID ∆T0-T12m	29 (91%)	33 (85%)
	PASS T3m	9 (26%)	9 (24%)
	PASS T12m	24 (71%)	26 (67%)
KOOS, QoL	T0	22.9 ± 8.1	21.5 ± 8.5
	T6w	39.1 ± 13.0	47.8 ± 19.1
	T3m	47.9 ± 21.0	50.2 ± 21.3
	T12m	64.7 ± 23.9	65.3 ± 20.3
	MCID ∆T0-T3m	22 (69%)	26 (68%)
	MCID ∆T0-T12m	27 (87%)	34 (87%)
	PASS T3m	7 (20%)	8 (21%)
	PASS T12m	17 (50%)	16 (41%)
KOOS, symptoms	T0	50.2 ± 10.4	49.6 ± 9.4
	T6w	56.0 ± 17.8	55.0 ± 12.8
	T3m	65.6 ± 17.5	61.9 ± 16.6
	T12m	77.7 ± 18.1	80.0 ± 14.2
	MCID ∆T0-T3m	22 (67%)	24 (63%)
	MCID ∆T0-T12m	25 (78%)	34 (87%)
	PASS T3m	9 (26%)	4 (11%)
	PASS T12m	18 (53%)	23 (59%)
VAS pain	T0	51.9 ± 10.1	51.2 ± 10.5
	T6w	34.6 ± 19.3	32.0 ± 18.3
	T3m	29.7 ± 25.8	24.2 ± 19.9
	T12m	14.3 ± 19.7	14.1 ± 19.0
	MCID ∆T0-T3m	18 (55%)	21 (58%)
	MCID ∆T0-T12m	25 (78%)	27 (71%)
	PASS T3m	21 (60%)	22 (61%)
	PASS T12m	29 (85%)	32 (84%)

*n*, number of patients; KOOS, Knee injury and Osteoarthritis Outcome Score; ADL, activities of daily living; QoL, quality of life; VAS, visual analog scale for pain; w, weeks; m, months; MCID, minimal clinical important difference; PASS, patient acceptable symptom state

**Table 3:** Generalized estimating equation models^a^ of intervention effects on subjective outcomes

		Intervention (resection) vs. control (preservation)		
		B^b^	95% CI	*p*-value
Kujala	Overall	‒0.84	‒5.16, 3.48	0.703
	∆T0-T6w	‒3.42	‒10.45, 3.61	0.341
	∆T6w-T3m	‒0.57	‒8.39, 7.26	0.886
	∆T3m-T12m	‒1.08	‒9.79, 7.62	0.807
KOOS, ADL	Overall	‒2.03	‒6.51, 2.45	0.375
	∆T0-T6w	‒3.56	‒12.49, 5.38	0.435
	∆T6w-T3m	‒3.01	‒12.01, 6.00	0.513
	∆T3m-T12m	‒0.11	‒9.47, 9.69	0.982
KOOS, pain	Overall	‒0.77	‒5.86, 4.33	0.768
	∆T0-T6w	‒6.49	‒16.69, 3.72	0.213
	∆T6w-T3m	‒3.01	‒12.99, 6.97	0.555
	∆T3m-T12m	‒0.99	‒10.61, 8.63	0.840
KOOS, QoL	Overall	‒2.52	‒8.01, 2.98	0.369
	∆T0-T6w	**‒10.02**	**‒18.91**, **‒1.12**	0.027*
	∆T6w-T3m	‒3.69	‒14.32, 6.95	0.497
	∆T3m-T12m	‒1.92	‒13.49, 9.65	0.745
KOOS, symptoms	Overall	0.49	‒4.07, 5.04	0.835
	∆T0-T6w	0.41	‒8.96, 9.79	0.931
	∆T6w-T3m	3.11	‒6.80, 13.01	0.539
	∆T3m-T12m	‒2.94	‒12.54, 6.67	0.549
VAS pain	Overall	2.60	‒2.76, 7.96	0.320
	∆T0-T6w	1.89	‒8.20, 11.98	0.714
	∆T6w-T3m	4.84	‒7.43, 17.11	0.439
	∆T3m-T12m	‒0.39	‒11.85, 11.08	0.947

^a^ All models were adjusted for baseline values of the particular outcome measure. The control group was the reference group in all analyses

^b^ B, regression coefficient; represents the between-group difference, i.e. the intervention effect relative to the control group over all measurements (‘Overall’) or for the specified measurement interval

* *p* < .05

CI, confidence interval; KOOS, Knee injury and Osteoarthritis Outcome Score; ADL, activities of daily living; QoL, quality of life; VAS, visual analog scale for pain; w, weeks; m, months

### Intervention effects on objective outcomes

Range-of-motion and gait quality outcomes over time are shown in Table 4, and longitudinal analysis results are presented in Table 5. Concerning range-of-motion, knee flexion was worse at 3 months compared to baseline (active ‒9.3°, *p* < .001; passive ‒10.7°, *p* < .001) but subsequently improved to 12 months (active 8.9°, *p* < .001; passive 8.9°, *p* < .001) in all patients. For knee extension, only passively measured extension deteriorated in the first 3 months (‒1.8°, *p* = .011; active ‒0.5°, *p* = .563), but both improved to 12 months (active 4.0° *p* < .001; passive 4.3° *p* < .001) in all patients. There were no overall intervention effects, while one difference was found at a specific interval. Compared to the control group, the intervention group improved their passive knee extension more between baseline and 3 months (B = 3.02, 95% CI[0.45, 5.60], *p* = .021).

**Table 4:** Objective outcomes for intervention and control groups

		Intervention group (resection, *n* = 42)	Control group (preservation, *n* = 43)
Active knee flexion	T0	118.9 ± 5.6	118.0 ± 6.1
	T3m	107.4 ± 13.8	111.1 ± 12.3
	T12m	115.6 ± 12.7	120.4 ± 12.1
Passive knee flexion	T0	122.7 ± 5.9	121.9 ± 6.0
	T3m	110.0 ± 13.8	113.4 ± 12.1
	T12m	117.6 ± 12.9	123.2 ± 11.7
Active knee extension	T0	‒9.3 ± 2.9	‒8.6 ± 2.5
	T3m	‒8.6 ± 4.9	‒10.2 ± 3.9
	T12m	‒5.2 ± 3.3	‒5.6 ± 4.3
Passive knee extension	T0	‒5.9 ± 2.1	‒4.9 ± 2.3
	T3m	‒6.2 ± 4.2	‒8.3 ± 4.0
	T12m	‒2.6 ± 3.5	‒3.2 ± 4.3
10 m-PWS, m/s	T0	1.16 ± 0.03	1.15 ± 0.03
	T3m	1.19 ± 0.21	1.18 ± 0.29
	T12m	1.33 ± 0.22	1.34 ± 0.25
	MCID ∆T0-T3m	8 (29%)	8 (31%)
	MCID ∆T0-T12m	15 (52%)	24 (71%)
	PASS T3m	13 (42%)	15 (52%)
	PASS T12m	23 (74%)	27 (71%)
Gait speed, m/s	T0	1.16 ± 0.04	1.14 ± 0.04
	T12m	1.25 ± 0.17	1.31 ± 0.15
	MCID ∆T0-T12m	6 (40%)	14 (67%)
	PASS T12m	15 (56%)	16 (62%)
Stride length, m	T0	1.31 ± 0.03	1.29 ± 0.03
	T12m	1.36 ± 0.15	1.38 ± 0.12
Gait symmetry (harmonic ratio AP)	T0	2.83 ± 0.21	2.75 ± 0.16
	T12m	3.00 ± 0.99	3.47 ± 1.06
Stride regularity (regularity ML)	T0	0.62 ± 0.02	0.61 ± 0.02
	T12m	0.62 ± 0.14	0.64 ± 0.13
Stride regularity (regularity VT)	T0	0.77 ± 0.02	0.76 ± 0.04
	T12m	0.77 ± 0.08	0.81 ± 0.10

*n*, number of patients; PWS, preferred walking speed; m, meters; s, seconds; AP, anterior-posterior; VT, vertical; ML, medial-lateral; m, months; MCID, minimal clinical important difference; PASS, patient acceptable symptom state

**Table 5:** Generalized estimating equation models^a^ of intervention effects on objective outcomes

		Intervention (resection) vs. control (preservation)		
		B^b^	95% CI	*p*-value
Active knee flexion	Overall	‒2.43	‒5.69, 0.83	0.144
	∆T0-T3m	‒4.52	‒12.76, 3.72	0.283
	∆T3m-T12m	‒5.78	‒13.03, 1.47	0.118
Passive knee flexion	Overall	‒2.75	‒6.05, 0.55	0.103
	∆T0-T3m	‒4.09	‒12.39, 4.20	0.333
	∆T3m-T12m	‒6.29	‒13.56, 0.98	0.090
Active knee extension	Overall	0.20	‒0.73, 1.12	0.678
	∆T0-T3m	2.40	‒0.62, 5.43	0.120
	∆T3m-T12m	1.19	‒1.47, 3.86	0.380
Passive knee extension	Overall	0.19	‒0.69, 1.07	0.670
	∆T0-T3m	3.02	0.45, 5.60	0.021*
	∆T3m-T12m	1.54	‒1.01, 4.09	0.237
10 m-PWS, m/s	Overall	0.01	‒0.07, 0.05	0.801
	∆T0-T3m	0.01	‒0.12, 0.14	0.912
	∆T3m-T12m	‒0.01	‒0.13, 0.10	0.855
Gait speed, m/s	Overall	‒0.02	‒0.06, 0.02	0.260
	∆T0-T12m	‒0.09	‒0.18, 0.01	0.071
Stride length, m	Overall	‒0.01	‒0.03, 0.03	0.951
	∆T0-T12m	‒0.03	‒0.12, 0.05	0.421
Gait symmetry (harmonic ratio AP)	Overall	‒0.15	‒0.38, 0.08	0.206
	∆T0-T12m	‒0.56	‒1.14, 0.02	0.057
Stride regularity (regularity ML)	Overall	‒0.01	‒0.04, 0.02	0.712
	∆T0-T12m	‒0.03	‒0.10, 0.05	0.523
Stride regularity (regularity VT)	Overall	‒0.01	‒0.03, 0.01	0.242
	∆T0-T12m	‒0.06	‒0.12, 0.01	0.071

^a^ All models were adjusted for baseline values of the particular outcome measure. The control group was the reference group in all analyses

^b^ B, regression coefficient; represents the between-group difference, i.e. the intervention effect relative to the control group over all measurements (‘Overall’) or for the specified measurement interval

* *p* < .05

CI, confidence interval; PWS, preferred walking speed; m, meters; s, seconds; AP, anterior-posterior; VT, vertical; ML, medial-lateral; m, months

Regarding gait, all patients had steady 10 m-PWS between baseline and 3 months but improved their 10 m-PWS from 3 to 12 months (with 0.15 m/s, *p* < .001). Instrumented gait speed (0.13 m/s, *p* < .001), stride length (0.08 m, *p* < .001), symmetry (0.49, *p* = .002), and regularity VT (0.03, *p* = .041) improved from baseline to 12 months, but not regularity ML (0.02, *p* = .393). There were no overall intervention effects or effects at specific intervals for any of the gait parameters. Clinical improvement in 10 m-PWS and gait speed appeared more often achieved at 12 months in the control group (MCID ∆T0-T12m: 52% versus 71%, *p* = .124, and 40% versus 67%, *p* = .112, respectively), however, these differences were not statistically significant. No differences in PASS were noted between groups (Table 4).

### Subjective versus objective outcomes

PROMs showed substantial improvement in the first 3 months postoperative, while objective outcomes improved between 3 and 12 months. Few moderate correlations were found between the change scores from baseline to 3 months (Table 6). In the intervention group, change in active knee flexion was moderately to strongly correlated with change in Kujala and all KOOS subscales, while in the control group, this was only associated with change in KOOS symptoms. Intervention patients also showed moderate associations between change in knee extension and KOOS symptoms, whereas control patients did not. Contrary, in the control group, change in 10 m-PWS was moderately correlated with change in VAS pain (*r*=‒.40) while no such association was found in the intervention group. Change in the 10 m-PWS from 3 to 12 months was moderately to strongly correlated with change in VAS, Kujala, KOOS ADL, and KOOS pain in the control group but not in the intervention group (Table 7). Finally, from baseline to 12 months, intervention patients showed mainly strong associations between change in 10 m-PWS, gait speed, cadence, stride length and change in Kujala and KOOS subscales, whereas control patients did not (Table 8). Change in knee flexion was moderately to strongly correlated with change in Kujala and all KOOS subscales in the intervention group, while in the control group, this was only associated with change in KOOS symptoms.

**Table 6:** Pearson’s correlations between subjective and objective outcomes for change scores between baseline and 3 months (∆T0-T3m)

	VAS	Kujala	KOOS, ADL	KOOS, pain	KOOS, QoL	KOOS, symptoms
*Intervention group*						
Active knee flexion	‒0.155	0.660**	0.448*	0.513*	0.415*	0.641**
Passive knee flexion	0.017	0.641**	0.348	0.334	0.180	0.549**
Active knee extension	0.188	0.328	0.257	0.133	0.136	0.523*
Passive knee extension	0.011	0.151	0.154	0.139	0.127	0.419*
10 m-PWS	‒0.064	0.122	0.276	0.350	0.167	0.048
*Control group*						
Active knee flexion	‒0.032	0.417	0.178	0.391	0.090	0.542*
Passive knee flexion	0.045	0.262	0.177	0.456*	0.036	0.621**
Active knee extension	‒0.033	0.035	0.043	0.373	‒0.090	0.302
Passive knee extension	‒0.083	0.104	0.106	0.507*	0.047	0.282
10 m-PWS	‒0.439*	0.357	0.393	0.169	0.431*	0.091

VAS, visual analog scale for pain; KOOS, Knee injury and Osteoarthritis Outcome Score; ADL, activities of daily living; QoL, quality of life; PWS, preferred walking speed.

* *p* < .05, ** *p* < .01

**Table 7:** Pearson’s correlations between subjective and objective outcomes for change scores between 3 and 12 months (∆T3m-T12m)

	VAS	Kujala	KOOS, ADL	KOOS, pain	KOOS, QoL	KOOS, symptoms
*Intervention group*						
Active knee flexion	0.038	0.484*	0.159	0.193	0.328	0.512*
Passive knee flexion	0.090	0.365	0.129	0.223	0.377	0.190
Active knee extension	0.004	‒0.126	‒0.036	‒0.268	‒0.379	0.005
Passive knee extension	0.204	‒0.229	‒0.136	‒0.090	‒0.164	0.133
10 m-PWS	0.370	‒0.231	‒0.008	‒0.318	‒0.279	‒0.137
*Control group*						
Active knee flexion	‒0.333	0.418	0.267	0.268	0.322	0.310
Passive knee flexion	‒0.286	0.314	0.352	0.372	0.518*	0.531*
Active knee extension	0.110	0.144	‒0.106	0.036	0.125	0.071
Passive knee extension	‒0.258	0.206	‒0.166	‒0.022	0.028	‒0.032
10 m-PWS	‒0.567**	0.574**	0.476*	0.402*	0.212	0.304

VAS, visual analog scale for pain; KOOS, Knee injury and Osteoarthritis Outcome Score; ADL, activities of daily living; QoL, quality of life; PWS, preferred walking speed.

* *p* < .05, ** *p* < .01

**Table 8:** Pearson’s correlations between subjective and objective outcomes for change scores between baseline and 12 months (∆T0-T12m)

	VAS	Kujala	KOOS, ADL	KOOS, pain	KOOS, QoL	KOOS, symptoms
*Intervention group*						
Active knee flexion	‒0.223	0.566**	0.514**	0.571**	0.568**	0.644**
Passive knee flexion	‒0.044	0.477*	0.413*	0.512**	0.485*	0.574**
Active knee extension	‒0.259	0.335	0.461*	0.453*	0.310	0.572**
Passive knee extension	‒0.164	0.113	0.168	0.153	0.064	0.292
10 m-PWS	‒0.339	0.514**	0.555**	0.555**	0.295	0.447*
Gait speed	‒0.732**	0.787**	0.862**	0.889**	0.691**	0.823**
Cadence	‒0.505	0.641*	0.645*	0.661*	0.577*	0.642*
Stride length	‒0.644*	0.671**	0.764**	0.793**	0.601*	0.712**
Gait symmetry (harmonic ratio AP)	0.061	‒0.313	‒0.237	‒0.263	‒0.224	‒0.334
Stride regularity (regularity ML)	‒0.004	0.492	0.368	0.342	0.473	0.501
Stride regularity (regularity VT)	‒0.262	0.312	0.405	0.267	0.516	0.312
*Control group*						
Active knee flexion	0.011	0.136	0.130	0.222	0.107	0.338
Passive knee flexion	0.089	0.233	0.100	0.217	0.149	0.408*
Active knee extension	‒0.226	0.249	0.127	0.407*	0.134	0.139
Passive knee extension	‒0.082	0.090	‒0.045	0.272	0.022	‒0.132
10 m-PWS	‒0.378*	0.085	0.380*	0.316	0.113	0.110
Gait speed	‒0.098	‒0.102	‒0.084	0.336	0.020	0.086
Cadence	0.145	‒0.353	‒0.261	‒0.049	‒0.096	‒0.017
Stride length	‒0.199	0.051	‒0.032	0.507*	0.059	0.083
Gait symmetry (harmonic ratio AP)	‒0.091	0.246	0.103	‒0.136	0.310	‒0.110
Stride regularity (regularity ML)	0.301	‒0.105	‒0.331	‒0.135	‒0.295	‒0.137
Stride regularity (regularity VT)	0.443*	‒0.325	‒0.567**	‒0.185	‒0.210	‒0.183

VAS, visual analog scale for pain; KOOS, Knee injury and Osteoarthritis Outcome Score; ADL, activities of daily living; QoL, quality of life; PWS, preferred walking speed; AP, anterior-posterior; VT, vertical; ML, medial-lateral

* *p* < .05, ** *p* < .01

## Discussion

This study aimed to compare the functional recovery and outcomes between TKA patients with or without Hoffa’s fat pad resection. Despite both groups showing significant improvements up to one year post-surgery, no significant differences between the groups were found for any of the functional outcomes over time. Clinical improvement was comparable between the resection and preservation group, and similar proportions of patients reached the PASS for subjective function and gait speed. Finally, objective outcomes showed a delayed recovery pattern compared to subjective outcomes, with changes in knee flexion and gait quality being moderately to strongly correlated with subjective function in resection but not in preservation patients.

In this study, functional outcome was assessed using both subjective as well as objective measurement tools. We found no differences between the groups throughout the follow-up period, except for one subscale of the KOOS at 6 weeks in favor of the preservation group. These findings are consistent with earlier comparative studies that also showed similar functioning for resection and preservation patients [2, 6]. However, all these studies utilized the Knee Society Score (KSS) for that purpose. Although the KSS has been the most popular method of reporting outcomes after knee arthroplasty worldwide [28], compared to the KOOS it insufficiently measures symptoms and daily activities from the patients’ perspective. Moreover, the KOOS was developed to measure the expectations of younger and more active patients, and as such fits better with the current population of TKA patients. A recent comparative study by Michalak et al. also used the KOOS to measure short-term functional outcome after either resection or preservation of the fat pad and concluded that there were no differences on all KOOS subscales up to 6 months postoperatively [29]. Considering other sortlike instruments, Walker et al. recently performed a meta-analysis on Oxford Knee Scores and showed mixed results [8]. Although statistically the preservation patients reported superior outcomes at 6 months post-surgery, clinically this effect was negligible. A common limitation of these studies and the current study is the follow-up of maximum one year post-surgery. However, one year is generally accepted as endpoint of recovery, and PROMs scores have been shown to not further improve beyond this time point [30]. Based on the present and earlier evidence, the lack of differences between the two interventions suggests that recovery of subjective function is not affected by resecting Hoffa’s fat pad.

With increasing use of PROMs in arthroplasty research and increasing understanding of their properties, several limitations have also come to light. Many have argued that PROMs alone are not enough to supply a complete overview of functional outcome. Over the last few years, there has been increasing interest in adopting performance tests and gait analysis to evaluate functional outcomes after TKA [12, 13]. Specifically, gait speed is considered a sensitive and meaningful parameter of functional recovery [14]. To our knowledge, this was the first RCT assessing the effect of fat pad resection compared to preservation on gait parameters. From a timed 10 m walk test we were able to determine the patients’ PWS. Both groups showed a similar significant improvement in 10 m-PWS, mainly gained between 3- and 12 months post-surgery. Furthermore, gait analysis using an inertial sensor provided even more accurate measures of gait speed and quality measures that could be improved after TKA [15]. We consequently found significant improvements in gait speed, stride length, and symmetry, without any meaningful differences between the groups. This lack of differences in gait outcomes should be considered as surgeons decide to resect or preserve the fat pad during TKA surgery. The present findings suggest that each of the surgical interventions result in similar – objectively measured – functional outcomes. But again, the current follow-up length must be taken into account as joint loading may further restore beyond this point.

Interestingly, in both groups, around 60–70% of patients reached PASS for gait speed at 12 months follow-up, with an overall average of 1.33 m/s on the 10 m-PWS and 1.27 m/s using the inertial sensor. Preoperatively, the average gait speed was already around 1.15 m/s in both groups which is fairly higher then previously reported in TKA populations. Other studies showed gait speeds improving from 0.99 to 1.06 m/s preoperative to 1.10–1.23 m/s one year postoperative in TKA patients [15, 16]. In healthy controls of 60–70 years old, the normal walking speed is 1.2 m/s for women and 1.4 m/s for men [31]. As such, the gait speeds achieved postoperatively by our cohort are close to normal but higher compared to TKA literature. This contrasts with earlier studies that reported remaining gait differences between healty controls and patients one year after TKA [16, 32]. An important consideration is that the current instrumented gait measurements were performed while patients were actively monitored by a researcher, which may have enabled them to achieve high levels of gait quality (known as the Hawthorne effect). Moreover, in the 10 m-PWS test, patients were allowed to get up to speed before being timed. These details in measuring gait may explain why our study population walked relatively fast both prior to and after TKA.

Based on recent literature [14–16], we could have expected to find different recovery patterns and only weak associations between PROMs and gait-related objective measures. We found that patient-reported pain and function greatly improved within the first 3 postoperative months, while range-of-motion and walking speed remained at or even decreased compared to baseline levels. These inverse recovery patterns in the early postoperative phase, with improving PROMs compared to worsening performance test scores, have also been reported by others [14, 16, 33]. Interestingly, we noted moderate to strong correlations between change scores in subjective and objective measures in resection patients, but not in preservation patients. Between baseline and one year post-surgery, changes in knee flexion and (instrumented) gait speed in resection patients were associated with changes in the Kujala and all KOOS subscales. In other words, improvement in knee flexion and gait speed indicated better self-reported functioning. Why these correlations were non-existent in preservation patients is difficult to clarify, as both groups were functioning highly comparable postoperatively. Perhaps other clinical outcomes not included in the present study could explain this inequality in presence or absence of correlations. For example, multiple meta-analyses have shown that patella tendon length is shortened in the resection group [4, 8, 34], which may subsequently impair knee flexion and increase postoperative pain. This explanation, however, does not align with current results and therefore does not seem likely. Alternatively, patient expectations, satisfaction, or other perceived outcomes – which also correlate with gait speed and knee flexion [26] – could play a role. Finally, it is also conceivable that these inequalities are a statistical coincidence and that the truth lies in the middle. After all, multiple studies have reported that for TKA patients in general, recovery of self-reported function and gait is only moderately associated [13–16, 34].

This study has some limitations, despite the design of a double-blind randomized controlled trial, the adoption of modern measurement tools, and the low dropout rate. A main limitation was the missing data (20–32%) of especially the objective outcomes. During the study we encountered a turnover of research personnel which interfered with completion of study actions for some patients. Regardless, most did complete all measurements, and PROMs response was satisfactory. Moreover, objective measurement of physical function was limited to knee range-of-motion and straight-on gait, while the subjective measurements included a range of daily life activities. Other performance tests such as a timed up-and-go, stair climbing, or isokinetic strength testing would have provided additional insights. Furthermoe, this study describes a short-term (one-year post-surgery) evaluation in which results may alter on the long run. Finally, one should be careful to draw strong conclusions from single significant findings due to multiple testing. Regardless, these limitations are not considered to have any direct effects on the results of this study.

## Conclusions

In this double-blind, randomized, controlled trial, patients who had Hoffa’s fat pad resected during TKA showed similar short-term functional outcomes as those who had their fat pad preserved. Surgeons should decide to keep or resect the fat pad merely based on the surgical exposure and not on functional outcomes that will be achieved in the first postoperative year. However, longer follow-up studies are needed to confirm these conclusions maintain at long-term follow-up.
